# Prevalence of Elevated Alanine Aminotransferase by Diagnostic Criterion, Age, and Gender among Adolescents

**DOI:** 10.1155/2020/4240380

**Published:** 2020-01-25

**Authors:** Jing Zhang, Zheng-Ying Wang, Jing-Ping Zhang, Hua Zhou, Zan Ding

**Affiliations:** ^1^Department of Nursing, Baoan Central Hospital of Shenzhen, The Fifth Affiliated Hospital of Shenzhen University, Shenzhen, Guangdong 518102, China; ^2^The Institute of Nursing Psychological Research Center, Xiangya College of Nursing, Central South University, Changsha, Hunan 410013, China; ^3^Institute of Low Carb Medicine, Baoan Central Hospital of Shenzhen, The Fifth Affiliated Hospital of Shenzhen University, Shenzhen, Guangdong 518102, China

## Abstract

**Background:**

Serum alanine aminotransferase (ALT) activity was measured not only to detect liver disease, but also to monitor overall health. The purpose of this study was to obtain the prevalence of elevated ALT levels among adolescents.

**Methods:**

In a school-based cross-sectional study, a representative sample was analyzed from 9 middle and high schools in Shenzhen, China, during 2017 to 2018. Elevated ALT was defined as diagnostic criterion I (>30 U/L for boys and >19 U/L for girls) and diagnostic criterion II (>40 U/L).

**Results:**

From the adolescent population, a total of 7281 students (boys, 4014, and girls, 3267) aged from 10 to 17 years were collected. The prevalence of elevated ALT was 7.11% (6.88% for boys and 7.41% for girls) by criterion I and 2.72% (3.96% for boys and 1.19% for girls) by criterion II. Based on the Shenzhen census and Chinese national census population, the adjusted prevalence of elevated ALT was 7.65% (boys 7.19% and girls 8.21%) and 6.79% (boys 6.07% and girls 7.56%) by criterion I and 2.85% (boys 4.20% and girls 1.16%) and 2.43% (boys 3.49% and girls 1.29%) by criterion II. For age, the overall trends were increasing progressively, regardless of the use of diagnostic criteria for an elevated ALT activity.

**Conclusions:**

This study supplements the gap that the prevalence of elevated ALT levels differed in gender, age, and criteria among adolescents of Shenzhen. We should take the prevalence as a predictor and continue to play a warning and preventive role in preparation for further intervention.

## 1. Introduction

Serum alanine aminotransferase (ALT) is a liver enzyme in the cytosol of hepatocytes, and the elevated values are frequently used to evaluate liver dysfunction [1]. As an indicator of health and disease [2], ALT has been proven several times to be a sensitive biochemical marker to screen for the detection of possible diseases, such as cardiovascular complications [3], osteopenia, bone mineral density [4], obstructive sleep apnea-hypopnea syndrome [5], anorexia nervosa [6], and depression [7]. As a part of annual check-ups, ALT levels increased after different kinds of liver injuries and when at risk of hepatitis, liver cirrhosis, liver steatosis, nonalcoholic steatohepatitis, hepatic hypoperfusion, and nonalcoholic fatty liver disease (NAFLD), which were more common in individuals with elevated ALT than with normal ALT [8, 9]. There were different causes of elevated ALT, including obesity-related diseases, hypertension, hyperglycemia, hypertriglyceridemia, diabetes, medication use, and metabolic syndrome [10, 11]. It is necessary to evaluate the prevalence of elevated ALT, which could be used as one of the surrogate markers of overall health in a general population.

Numerous national studies found that the prevalence of elevated ALT levels among adults and adolescents varied from different countries, ethnic groups, gender, age, and different diagnostic criteria [12–16]. Among adolescents, the prevalence of elevated ALT was shown in the U.S. [17–20], Korea [12, 21], Mexico [22, 23], China [10], Netherlands [24], and Iran [25]. The considerably different prevalence had pointed out the importance of standardizing and documenting the variations of ALT levels. Moreover, adolescents have been one of the group of greatest concern to society, because due to rapid growth at youth, the abnormalALT elevation may have further influence among adolescents than adults in the future for overall health [1, 26].

However, the prevalence of elevated ALT among adolescents had not been adequately evaluated in mainland China, especially in the new metropolis of Shenzhen. As one of the super bigger and newest first-tier cities worldwide, the total area of Shenzhen was 1997.3 km^2^ and the population was around 11.37 million with about 70% being migrants. Therefore, we really need to implement a clinical survey and to analyze the prevalence of elevated ALT among the adolescents of Shenzhen, China, in a cross-sectional study. The objectives of the present study were to examine the crude and standardized prevalence of elevated ALT for the total adolescent group as well as the subgroups by age and gender and to summarize the prevalence of elevated ALT among adolescents all over the world.

## 2. Materials and Methods

### 2.1. Study Population

The current study was conducted in Shenzhen, located in the Pearl River Delta region in southern China. As the first special economic zone of China and one of the largest manufacturing bases in the world, Shenzhen has a total gross domestic product (GDP) of 0.27 trillion USD and 24,640 USD per capita in 2015.

We implemented a school-based descriptive survey and recruited all the freshmen from 9 junior and senior high schools (HangCheng School, Baoan Vocational Technical School, Oriental English School, HeZhou School, HuangMaBu School, JinBi Experimental School, KangQiao School, LongShan School, and ZhongAo Experimental School) [27]. The whole clinical data were extracted from the compulsory admission physical examination organized by the Baoan District Government of Shenzhen from February 2017 to June 2018. In this study, adolescents were defined as 10–17 years old. A total of 7382 students initially participated in our study; 28 cases ≥ 18 years old, 16 cases < 10 years old, 41 cases lacked ALT, and 16 cases with missed age were deleted. Finally, 7281 valid samples remained (effective rate 98.6%).

### 2.2. Data Collection

Demographic information such as age, sex, and grade were obtained from self-administered questionnaires. Anthropometric and laboratory measurements of students were also taken. Height and weight were measured by trained physicians to the nearest 0.5 cm and 0.1 kg, without shoes, socks, and any other heavy clothing. After an overnight fast of 10 h, blood samples were drawn from an antecubital vein in each subject into vacutainer tubes. The tubes were delivered to the laboratory of the hospital in an insulated box with ice by a specially assigned physician and centrifuged within an hour. ALT was measured using the AU5800 serial with the automatic biochemical analyzer (Model AU5821; Tokyo, Japan) by the manufacturing enterprise Beckman Coulter K.K. Written informed consents for each participant and their parents were obtained.

### 2.3. Definition of Elevated Serum ALT Levels

For adolescents, the common definitions of elevated ALT used were above 30 U/L for boys and above 19 U/L for girls [25, 28]. Besides, ALT values were quantitatively estimated above 40 U/L in previous studies [19, 22]. In the present study, the prevalence of elevated ALT was mainly diagnosed by ALT > 30 U/L for boys and >19 U/L for girls (i.e., criterion I) and ALT > 40 U/L for both sexes (i.e., criterion II).

### 2.4. Statistical Analysis

The normal distribution of serum ALT was determined by the Shapiro-Wilk test. Significance for intergroup differences was evaluated by the Mann-Whitney *U* test for the skewed blood index. We combined 10–11-year-old adolescents in the statistical analysis of the ALT abnormality rate, because the sample size of the 10 year olds was too small. The prevalence of elevated ALT with 95% confidence interval (95% CI) was quantitatively estimated, diagnosed by both criteria I and II. Significance for intergroup difference between boys and girls in the prevalence of elevated ALT was assessed by Pearson's chi-square test. A chi-square test for the trend of the crude age-prevalence of elevated ALT was also performed. Age- and/or sex-standardized prevalence rates of elevated ALT were estimated by the direct standardization method, according to the distribution of the population in the 2010 Shenzhen population census (http://www.sztj.gov.cn/xxgk/zfxxgkml/tjsj) as well as the 2010 Chinese population census (http://www.stats.gov.cn/tjsj). Associations with age and gender were examined using multiple logistic regression models, expressed as odds ratios and 95% CIs for risk of elevated ALT.

All statistical analyses involved use of SPSS for Windows 13.0 (SPSS Inc., Chicago, IL, USA) and R software version 3.2.0 (http://www.R-project.org, the R Foundation for Statistical Computing, Vienna, Austria), and graphics were carried out by SigmaPlot software version 10.0. Two-tailed tests of significance were reported, and *P* < 0.05 was considered statistically significant.

## 3. Results

### 3.1. Sample Characteristics

Overall, we collected 7281 adolescents aged 10–17 years for analysis, involving 4014 boys and 3267 girls.  Table 1 shows the baseline descriptive statistics for the levels of ALT stratified by age and gender. For 10–17 year olds, the mean (standard deviation) value of ALT was separately 15.96 (17.02), 12.18 (11.06), and 14.26 (14.77) U/L for boys, girls, and both genders, respectively. Boys had higher ALT levels than girls (*P* < 0.001). The concentrations of serum ALT were not normally distributed (*P* < 0.001). The 5th, 25th, 75th, and 95th percentiles of serum ALT levels were 7, 9, 15, and 30 U/L for overall participants; 7, 10, 16, and 37 U/L for boys; and 6, 9, 13, and 22 U/L for girls, respectively.

### 3.2. Crude Prevalence of Elevated ALT

**Figure 1:**
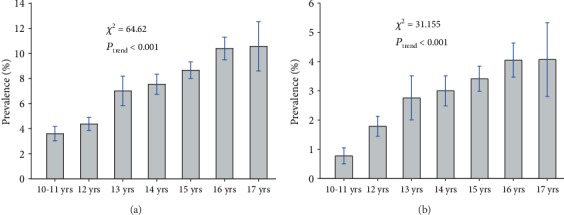
Crude age-prevalence of elevated alanine aminotransferase (ALT) based on the diagnostic criteria I (a) and II (b) among the adolescents of Shenzhen, China, 2017–2018.

As shown in Table S1, a total of 518 and 198 adolescents had abnormal ALT levels separately, based on the diagnostic criteria I (>30 U/L for boys and >19 U/L for girls) and II (>40 U/L). The crude age-prevalence of elevated ALT among adolescents was 7.11% (6.88% for boys and 7.41% for girls) by criterion I and 2.72% (3.96% for boys and 1.19% for girls) by criterion II. Obviously, the prevalence of elevated ALT, influenced by gender and age, was higher by criterion I than by criterion II. In addition, Figure 1 shows the trends of the crude age-prevalence of elevated ALT, which displayed an uptrend by age (both *P* < 0.001).

### 3.3. Standardized Prevalence of Elevated ALT

**Figure 2:**
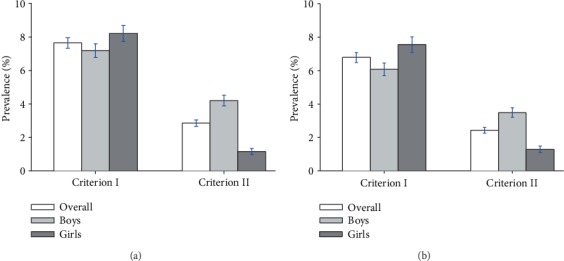
Standardized prevalence of elevated alanine aminotransferase (ALT) among the adolescents of Shenzhen, China, 2017–2018, diagnosed with both the criteria I and II, based on the 2010 Shenzhen Census Population (a) and the 2010 Chinese National Census Population (b).

According to the Shenzhen census, the overall standardized prevalence of elevated ALT was 7.65% (boys 7.19% and girls 8.21%) by criterion I and 2.85% (boys 4.20% and girls 1.16%) by criterion II; based on the Chinese national census, the standardized prevalence was 6.79% (boys 6.07% and girls 7.56%) by criterion I and 2.43% (boys 3.49% and girls 1.29%) by criterion II (Figure 2). The standardized prevalence of elevated ALT was similar with the results of crude prevalence shown in Table 2.

### 3.4. Univariable and Multivariable Logistic Regression Analyses

Tables 3 and 4 show the outcomes of gender and age difference with elevated ALT by logistic regression analysis. For gender, the abnormal rate of ALT in girls was much higher than that in boys (OR = 1.65, 95% CI: 1.35–2.03) by criterion I, but lower in girls (0.42, 0.29–0.60) by criterion II, after being adjusted for age and body mass index using a multivariable logistic regression. For age, compared to the reference group, the overall trends were increasing progressively, regardless of the use of diagnostic criteria for an elevated ALT activity.

### 3.5. The Prevalence of Elevated ALT among Adolescents Worldwide

In Table 5, we summarized the prevalence of elevated ALT among adolescents worldwide. The prevalence of elevated ALT among adolescents differed by the sex ratio and age of objects, area, ethnicity, study period, and diagnostic criterion [19, 22, 23, 29–31].

## 4. Discussion

Shenzhen is a vibrant city with a large number of immigrants, and the majority of the population here lives in a fast-paced and rapid-developing social environment [32]. This study examined the crude and standardized prevalence of elevated ALT among adolescents of Shenzhen for the total group and subgroups, showing that the prevalence of elevated ALT increased with age and differed by gender at puberty. The levels of ALT may be influenced by different variations including region, ethnic groups, overweight, obesity, liver diseases, and other factors, and we therefore summarized the prevalence of elevated ALT among adolescents on a global scale.

### 4.1. Diagnostic Criteria for Elevated ALT Levels

Nowadays, almost all countries use gender-specific criteria to determine the elevated ALT levels rather than the single standard usually used [18, 21, 33]. Our results obtained the prevalence of elevated ALT for boys and girls in diagnostic criteria I (>30 U/L for boys and >19 U/L for girls) and II (>40 U/L). Aside from the two main criteria above used in our study, there were other criteria (e.g., ALT > 33 U/L for boys and >25 U/L for girls [33], >30 U/L for boys and >21 U/L for girls [34], and >30 U/L for both sexes [24]) with which to diagnose an elevated ALT activity.

### 4.2. The Prevalence of Elevated ALT

Based on various criteria, the abnormal rates of elevated ALT obviously differed. Among the adolescents of Shenzhen, the prevalence of elevated ALT was 7.11% by criterion I and 2.72% by criterion II; in Korea, 7.9% for boys and 10.2% for girls by criterion I [28] and 3.6% for boys and 2.8% for girls by criterion II [12]; and in Hong Kong of China, 3.2% by ALT > 40 U/L and 5.9% by ALT > 30 U/L [35].

From Table 5, the prevalence of elevated ALT among adolescents ranged from 1.5% in mainland China for girls [10] to 34.9% in Korea for obese populations [33]. The prevalence of elevated ALT is diverse in different national groups, such as 4.9% for boys and 7.5% for girls in Iran by criterion I [25], 3.0% in Mexico (>40 U/L) [22], 8.0% in the U.S. (>30 U/L) [19], 3.1% in China (≥40 U/L) [10], and 5.3% (>30 U/L) and 2.8% (>40 U/L) in Korea [34].

### 4.3. Prevalence of Elevated ALT Differed by Gender

Gender differences turned up in our study; for example, the proportion of the abnormal rate was higher for girls than boys by criterion I (7.41% vs. 6.88%, respectively) but lower by criterion II (1.19% vs. 3.96%, respectively). Evaluating the abnormal ALT levels by gender shows more individuality than gender-neutral assessment. Most studies reported different prevalence rates of elevated ALT by gender (e.g., 8.0% for boys and 2.1% for girls in Korea [21], 5.6% for boys and 1.6% for girls in the U.S. [19], 4.8% for boys and 1.5% for girls in mainland China [10], and 9.8% for boys and 3.8% for girls in Mexico [23]).

The levels of serum ALT were higher in boys than in girls, which may relate to the differences in muscle mass and endogenous hormone levels (self-undetermined physical and emotional factors) between genders [36–39]. In this regard, it is well acknowledged that serum concentrations of estradiol are low in preadolescent girls and increase at menarche [40, 41]. The age-related hormonal changes could partly account for the ALT levels that we observed between genders.

### 4.4. Prevalence of Elevated ALT Differed by Age

With the metabolic liver diseases gradually becoming younger currently, the abnormal rates of elevated ALT clearly reflected an uptrend with growing age. Focused on the Shenzhen adolescents of 10–17 years, the prevalence of elevated ALT was a rising tendency with increasing age, regardless of the use of criterion I or II. For adults, the Rancho Bernardo Study cohort showed that the levels of ALT were highest in a young group (30–62 years) and subsequently decreased with increasing age among 63–93 years [16]; another longitudinal study also reported similar findings that the mean levels of ALT decreased with age in elders, with a mean age of 65.7 years [29]. From the above two studies, we could know the downtrend with age of ALT levels in the elderly age range. Containing 335 subjects (≥18 years), a Jerusalem study on age-ALT association reported the peaking age (40–55 years) from an inverted *U* curve [15]. Thus, uptrend with age in the adolescent period should probably be taken into account.

### 4.5. Other Influencing Factors for Abnormal ALT Levels

Except for the above four factors (i.e., diagnostic criterion, age, gender, and region), ethnicity may be a potential influencing factor to affect the prevalence of elevated ALT levels. It was significant to note that ethnics affect the abnormal rate in several studies (e.g., 13.7% in Hispanics, 8.6% in non-Hispanic whites, and 5.4% in non-Hispanic blacks [42]; 7.4% among whites, 6.0% among blacks, 11.5% in Mexican Americans, and 10.6% in multiracial and other Hispanic adolescents [19]).

It has been reported that ALT levels and the prevalence of elevated ALT were more common in overweight or obese adolescents (e.g., 6% of the overweight and 20% of the obese in the U.S. [20], 15.7% of the overweight and 34.9% of the obese in Korea [16], and 14.2% of the overweight and 28.9% of the obese in Mexico [33]).

The levels of ALT were closely connected with the damage of hepatocyte and hepatic enzymes. Elevated ALT played a predictive role in the diagnosis of liver diseases (e.g., NAFLD and metabolic liver diseases), which were prevalent but concealed in adolescents [43]. Furthermore, bad living habits (e.g., staying up late, lack of exercise, and increasingly sedentary lifestyle), unhealthy eating behaviors (e.g., drinking beverage, snacking, and take-away meal habits), and living settings were potentially important factors. From the above, healthy lifestyle and dietary intervention are crucial to weight loss and to keep adolescents healthy.

### 4.6. Limitations

Several potential limitations of our research should be acknowledged. First, participants were only from a single city of China, and it therefore might be difficult to generalize these findings for adolescents of other regions. Second, alcohol consumption and hepatitis B virus (HBV) and hepatitis C virus (HCV) infections were less common and out of our considerations [44, 45]. Third, the lack of comprehensive evaluation of individualized specific characteristics was not enough to conduct a further interventional study on the basis of this study.

## 5. Conclusions

The prevalence of elevated ALT levels differed in gender, age, and criteria among adolescents of Shenzhen. As the high growth period, adolescents have become a focus of public and national health; strengthening healthy education and lifestyle management may be benefits to their healthy growth. With timely attention to age and gender differences of adolescents during their earlier identification of elevated ALT, more adolescents may avoid the diagnosis of elevated ALT-related diseases, especially for boys and high school students. Consequently, we should take the prevalence as a predictor and continue to play a warning and preventive role in preparation for further intervention.

## Figures and Tables

**Table 1:** Baseline descriptive statistics for serum alanine aminotransferase levels (U/L) stratified by gender among the adolescents (aged 10–17 years) of Shenzhen, China, 2017–2018.

Sample	*N*	Mean	SD	Min	Percentiles								Max
					5th	10th	25th	50th	75th	90th	95th	99th	
Overall (years)													
10.0–10.9	60	11.25	5.04	3	6	7	9	10	13	14	15	33	39
11.0–11.9	967	12.23	6.85	3	7	8	9	11	14	17	20	38	141
12.0–12.9	1511	12.67	9.29	1	7	8	9	11	13	18	22	48	206
13.0–13.9	471	14.85	21.84	3	7	8	9	12	15	20	27	76	415
14.0–14.9	1101	14.21	12.76	1	7	8	9	11	14	22	33	68	193
15.0–15.9	1789	15.57	19.59	3	7	8	9	11	16	24	35	75	495
16.0–16.9	1136	15.80	15.54	3	7	7	9	12	16	25	36	81	213
17.0–17.9	246	15.29	11.38	4	7	8	9	12	16	25	33	65	93
10.0–17.9	7281	14.26	14.77	1	7	8	9	11	15	21	30	67	495
Boys (years)													
10.0–10.9	33	11.21	5.58	6	6	7	9	10	12	14	15	31	39
11.0–11.9	490	13.36	8.52	4	7	8	10	12	15	18	23	46	141
12.0–12.9	899	13.39	8.80	2	7	8	9	11	14	19	25	50	113
13.0–13.9	269	13.17	10.12	4	7	8	9	11	14	19	25	50	138
14.0–14.9	546	16.74	16.36	1	7	8	10	12	18	27	40	92	193
15.0–15.9	1017	18.23	24.31	3	7	8	10	13	18	29	40	116	495
16.0–16.9	624	18.51	19.45	3	7	8	10	13	19	33	50	102	213
17.0–17.9	136	17.16	13.62	4	7	8	10	12	18	32	49	65	93
10.0–17.9	4014	15.96	17.02	1	7	8	10	12	16	25	37	79	495
Girls (years)													
10.0–10.9	27	11.30	4.39	3	7	7	10	11	13	14	16	25	28
11.0–11.9	477	11.07	4.24	3	6	7	9	10	12	16	18	27	40
12.0–12.9	612	11.62	9.89	1	6	7	9	10	12	16	19	43	206
13.0–13.9	202	17.07	31.15	3	7	8	9	12	16	22	30	96	415
14.0–14.9	555	11.72	6.89	2	6	7	8	10	13	16	21	43	68
15.0–15.9	772	12.06	9.48	3	6	7	8	10	13	18	23	46	186
16.0–16.9	512	12.51	7.44	3	7	7	9	11	14	20	24	36	88
17.0–17.9	110	12.98	7.17	4	7	8	9	12	15	19	22	29	69
10.0–17.9	3267	12.18	11.06	1	6	7	9	10	13	17	22	46	415

ALT: alanine aminotransferase; SD: standard deviation.

**Table 2:** Crude age-prevalence of elevated alanine aminotransferase (ALT) stratified by gender among the adolescents of Shenzhen, China, 2017–2018, separately based on the diagnostic criteria I and II.

Age (years)	Criterion I^†^				Criterion II^‡^			
	Overall	Boys	Girls	*P* value^∗^	Overall	Boys	Girls	*P* value^∗^
10–17	7.11 (6.52-7.7)	6.88 (6.09-7.66)	7.41 (6.51-8.31)	0.380	2.72 (2.35-3.09)	3.96 (3.36-4.56)	1.19 (0.82-1.57)	**<0.001**
10–11	3.60 (2.46-4.74)	2.87 (1.44-4.30)	4.37 (2.58-6.15)	0.198	0.78 (0.24-1.32)	1.53 (0.48-2.58)	0 (0-0)	**0.005**
12	4.37 (3.34-5.40)	4.00 (2.72-5.29)	4.90 (3.19-6.61)	0.402	1.79 (1.12-2.45)	2.22 (1.26-3.19)	1.14 (0.30-1.99)	0.119
13	7.01 (4.70-9.31)	2.97 (0.94-5.00)	12.38 (7.83-16.92)	<0.001	2.76 (1.28-4.24)	1.49 (0.04-2.93)	4.46 (1.61-7.30)	**0.052**
14	7.54 (5.98-9.10)	8.24 (5.94-10.55)	6.85 (4.75-8.95)	0.381	3.00 (1.99-4.00)	4.76 (2.98-6.55)	1.26 (0.33-2.19)	**0.001**
15	8.66 (7.36-9.97)	8.95 (7.19-10.70)	8.29 (6.35-10.24)	0.624	3.41 (2.57-4.25)	5.01 (3.67-6.36)	1.30 (0.50-2.09)	**<0.001**
16	10.39 (8.61-12.16)	10.58 (8.16-12.99)	10.16 (7.54-12.77)	0.817	4.05 (2.90-5.20)	6.57 (4.63-8.51)	0.98 (0.12-1.83)	**<0.001**
17	10.57 (6.73-14.41)	11.03 (5.76-16.29)	10.00 (4.39-15.61)	0.794	4.07 (1.60-6.53)	6.62 (2.44-10.8)	0.91 (0-2.68)	**0.024**

The prevalence of elevated ALT is expressed as % (95% confidence interval). ^†^Diagnostic criterion I: >30 U/L for boys and >19 U/L for girls. ^‡^Diagnostic criterion II: >40 U/L for boys and girls. ^∗^*P* < 0.001 comparing girls with boys by Pearson's chi-square test.

**Table 3:** Univariate and multivariate logistic regression analyses with elevated alanine aminotransferase (ALT) as the outcome variable, based on the diagnostic criterion I (>30 U/L for boys and >19 U/L for girls).

Variable	Univariate analysis			Multivariate analysis^a^		
	Wald test	OR (95% CI)	*P*	Wald test	OR (95% CI)	*P*
Girls (vs. boys)	0.770	1.08 (0.91-1.30)	0.380	23.587	1.65 (1.35-2.03)	<0.001
Age (years)	62.625		<0.001	34.547		<0.001
10–11		1.00			1.00	
12	0.917	1.22 (0.81-1.84)	0.338	1.295	1.29 (0.83-2.01)	0.255
13	8.108	2.02 (1.24-3.27)	0.004	7.431	2.06 (1.23-3.47)	0.006
14	14.816	2.18 (1.47-3.25)	<0.001	5.601	1.68 (1.09-2.59)	0.018
15	24.716	2.54 (1.76-3.66)	<0.001	12.089	2.03 (1.36-3.02)	0.001
16	34.169	3.10 (2.12-4.53)	<0.001	19.535	2.53 (1.67-3.83)	<0.001
17	18.656	3.16 (1.88-5.33)	<0.001	15.973	3.17 (1.80-5.58)	<0.001
Constant	—	—	—	667.963	—	<0.001

Boys or 10–11 years as the reference group; OR (95% CI): odds ratio (95% confidence interval). ^a^Adjusted for gender, age, and body mass index using a multivariable logistic regression analysis.

**Table 4:** Univariate and multivariate logistic regression analyses with elevated alanine aminotransferase (ALT) as the outcome variable, based on the diagnostic criterion II (>40 U/L for boys and girls).

Variable	Univariate analysis			Multivariate analysis^a^		
	Wald test	OR (95% CI)	*P* value	Wald test	OR (95% CI)	*P* value
Girls (vs. boys)	46.388	0.29 (0.21-0.42)	<0.001	21.129	0.42 (0.29-0.60)	<0.001
Age (years)	28.711		<0.001	16.540		0.011
10–11		1.00			1.00	
12	4.315	2.32 (1.05-5.12)	0.038	3.872	2.32 (1.00-5.38)	0.049
13	8.054	3.62 (1.49-8.78)	0.005	9.492	4.31 (1.70-10.92)	0.002
14	11.940	3.94 (1.81-8.56)	0.001	7.945	3.24 (1.43-7.35)	0.005
15	15.808	4.50 (2.14-9.43)	<0.001	8.593	3.21 (1.47-6.99)	0.003
16	19.030	5.38 (2.53-11.44)	<0.001	11.086	3.90 (1.75-8.69)	0.001
17	12.346	5.40 (2.11-13.82)	<0.001	10.645	5.34 (1.95-14.60)	0.001
Constant	—	—	—	268.333	—	<0.001

Boys or 10–11 years as the reference group; OR (95% CI): odds ratio (95% confidence interval). ^a^Adjusted for gender, age, and body mass index using a multivariable logistic regression analysis.

**Table 5:** Summaries of the prevalence of elevated ALT among adolescents for serum alanine aminotransferase (ALT) worldwide.

No.	Study	Country	Target population	Age (years)	Sample size	Definition (U/L)	Prevalence of elevated ALT
1	Kim et al. [21] (2018)	Korea	Adolescents	10-18	8455	>30	5.3% in total 8.0% in boys 2.1% in girls
						>40	2.8% in total
						>33 for boys >25 for girls	5.4% in total

2	Ruhl and Everhart [18] (2013)	U.S.	Adolescents	12-19	9361	>31 for boys >24 for girls	10.8% am and 11.3% pm in boys 7.3% am and 7.8% pm in girls

3	Kong et al. [35] (2008)	China	Adolescents	12-18	2102	>40	3.2% in total
						>30	5.9% in total

4	Fraser et al. [19] (2007)	U.S.	Adolescents	12-19	5586	>30	8.0% in total 5.6% in boys 1.6% in girls 7.4% in white 11.5% in Mexican Americans 6.0% in black 10.6% in multiracial and other Hispanics
						>40	3.6% in total 5.6% in boys 1.6% in girls 3.1% in white 6.1% in Mexican Americans 2.3% in black

5	Strauss et al. [20] (2000)	U.S.	Overweight and obese adolescents	12-18	2450	>30	6% in overweight 20% in obesity

6	Park et al. [12] (2005)	Korea	Adolescents	10-19	1594	>40	3.6% in boys 2.8% in girls

7	Wu et al. [10] (2012)	China	Students	12-24	6997	≥40	3.1% in total 4.8% in boys 1.5% in girls

8	Park et al. [33] (2012)	Korea	Adolescents	12-18	1591	>33 for boys >25 for girls	5.9% in total 15.7% in overweight 34.9% in obesity

9	Fermin et al. [17] (2017)	U.S.	Adolescents	12-19	5411	>30	9.14% in total 8.74% in non-Hispanic white 5.49% in non-Hispanic black 13.33% in Hispanic
						>22 for girls >25 for boys	16.25% in total

10	DeBoer et al. [42] (2013)	U.S.	Adolescents	12-19	4124	>30	9.0% in total 13.7% in Hispanics 8.6% in non-Hispanic white 5.4% in non-Hispanic blacks

11	Ramirez-Lopez et al. [22] (2018)	Mexico	Adolescents	14-19	674	>40	3.0% in total 14.1% in obesity 1.81% in nonobesity

12	Purcell et al. [23] (2013)	Mexico	Youth	8-19	1262	>40	9.8% in boys 3.8% in girls 28.9% in obesity 14.2% in overweight 40.0% in metabolic syndrome boys 14.5% in metabolic syndrome girls

13	Elizondo-Montemayor et al. [32] (2014)	Mexico	Obese and overweight children	6-12	236	>40	17.7% in total

14	Samani et al. [25] (2011)	Iran	Children and adolescents	6-18	1172	>30 for boys >19 for girls	4.9% in boys 7.45% in girls

15	Wang et al. [43] (2018)	Korea	Healthy adolescents	10-18	1785	>24.1 for boys >17.7 for girls	14.9% in boys 12.3% in girls

16	van Vliet et al. [24] (2009)	Netherlands	Overweight and obese children	3-18	443	>30	20.3% in total 25.8% in boys 13.8% in girls

17	Park et al. [28] (2013)	Korea	Adolescents	10-18	2242	>30 for boys >19 for girls	7.9% in boys 10.2% in girls

## Data Availability

Readers can get the datasets of the current study by contacting the author of Jing Zhang via email (18002534959@163.com) for a reasonable request.

## Supplementary Materials

Supplementary Materials Table S1: number of students with elevated alanine aminotransferase (ALT) stratified by gender and age among the adolescents of Shenzhen, southern China, 2017–2018, separately based on the diagnostic criteria I and II.
