# Supplementary material for: Prevalence of Elevated Alanine Aminotransferase by Diagnostic Criterion, Age, and Gender among Adolescents
Source: Gastroenterol Res Pract. 2020 Jan 25;2020:4240380. doi: 10.1155/2020/4240380 (PMC7204184; doi:10.1155/2020/4240380)
Supplement: Supplementary Materials — Table S1: number of students with elevated alanine aminotransferase (ALT) stratified by gender and age among the adolescents of Shenzhen, southern China, 2017–2018, separately based on the diagnostic criteria I and II. [file 4240380.f1.doc]

**Table S1.** Number of students with elevated alanine aminotransferase (ALT) stratified by gender and age among the adolescents of Shenzhen, southern China, 2017−2018, separately based on the diagnostic criteria I and II.

| Age (years) | Criterion I^†^ | | |  | Criterion II^‡^ | | |
| --- | --- | --- | --- | --- | --- | --- | --- |
|  | Overall | Boys | Girls |  | Overall | Boys | Girls |
| 10−17 | 518 | 276 | 242 |  | 198 | 159 | 39 |
| 10−11 | 37 | 15 | 22 |  | 8 | 8 | 0 |
| 12 | 66 | 36 | 30 |  | 27 | 20 | 7 |
| 13 | 33 | 8 | 25 |  | 13 | 4 | 9 |
| 14 | 83 | 45 | 38 |  | 33 | 26 | 7 |
| 15 | 155 | 91 | 64 |  | 61 | 51 | 10 |
| 16 | 118 | 66 | 52 |  | 46 | 41 | 5 |
| 17 | 26 | 15 | 11 |  | 10 | 9 | 1 |

^†^ Diagnostic criterion I: >30U/L for boys and >19U/L for girls.

^‡^ Diagnostic criterion II: >40U/L for boys and girls.
